# A14 TRANSABDOMINAL BOWEL ULTRASOUND AND CLINICAL OUTCOMES OVER ONE YEAR IN CHILDREN WITH NEWLY DIAGNOSED CROHN’S DISEASE

**DOI:** 10.1093/jcag/gwac036.014

**Published:** 2023-03-07

**Authors:** A Hudson, D Isaac, K Novak, H Ma, A Kuc, M Carroll, E Wine, H Huynh

**Affiliations:** 1 Pediatric Gastroenterology, University of Alberta, Edmonton; 2 Gastroenterology, University of Calgary, Calgary, Canada

## Abstract

**Background:**

Transabdominal bowel ultrasound (TABUS) is an emerging non-invasive tool for monitoring inflammatory bowel disease (IBD). Its use is particularly increasing in pediatric IBD, given the need for anesthesia during endoscopy. The assessment of TABUS in pediatric IBD has been limited to small numbers of patients with no long-term follow-up.

**Purpose:**

To describe TABUS findings and its relationship with clinical, biochemical, and endoscopic assessments in pediatric patients with Crohn’s disease up to one year post-diagnosis.

**Method:**

Patients (0-18 years) with suspected IBD were prospectively enrolled through the Edmonton Pediatric IBD Clinic. Those with Crohn’s disease were included. Patients underwent repeated TABUS, clinical assessments, blood work, fecal calprotectin (FCP) (baseline, 1-, 3-, 6-, 12-months), and endoscopy (baseline and 6-12 months). The weighted Pediatric Crohn’s Disease Activity Index (wPCDAI), Simple Endoscopic Score for Crohn’s Disease (SES-CD; rectum excluded), and Simple Ultrasound Activity Score for Crohn’s Disease (SUS-CD; rectum excluded) were used. Remission was defined as FCP<250mg/kg, CRP<4mg/L, wPCDAI<12, no upcoming surgery, and SES-CD score ≤2 for any bowel segment.

**Result(s):**

Fifty-six patients (68% male), median age 12.5 years (range 6-17), were followed for 6 months. Forty (71%) were followed up to 12 months. Median TABUS bowel wall thickness (BWT) and SUS-CD total scores improved in all bowel segments over time. SUS-CD total scores significantly correlated with SES-CD (baseline, 6-, 12-months), wPCDAI (baseline, 1-, 6-, 12-months), CRP (baseline, 1-, 3-, 6-months), ESR (baseline, 1-, 3-, 6-, 12-months), and FCP (baseline, 1-, 6-, 12-months) (rho ranged 0.302-0.732, p<0.05). Patients in remission had sustained significantly thinner BWT of their most affected bowel segment (Figure 1) starting at 1-month (median 3.1mm (IQR 2.7-3.7) vs. 4.1mm (IQR 2.9-5.6; p<0.05), and sustained significantly lower SUS-CD total scores starting at 6 months (median 0 (IQR 0-1) vs. median 2 (IQR 1-3); p<0.05). Seven patients had surgery (n=7/7 ileocecal, n=2/7 jejunal resection). All 7 patients had complex TI disease (n=6 strictures, n=1 long-segment disease >25cm) and proximal small bowel disease (n=2/2) on TABUS. Those with baseline ultrasound findings of a stricture with upstream bowel dilatation (n=7/56) had increased odds (OR=288, p<0.01) and relative risk (RR=42, p<0.01) of needing surgery (n=6/7 with baseline obstructive findings, n=1/49 without) within the first year.

**Image:**

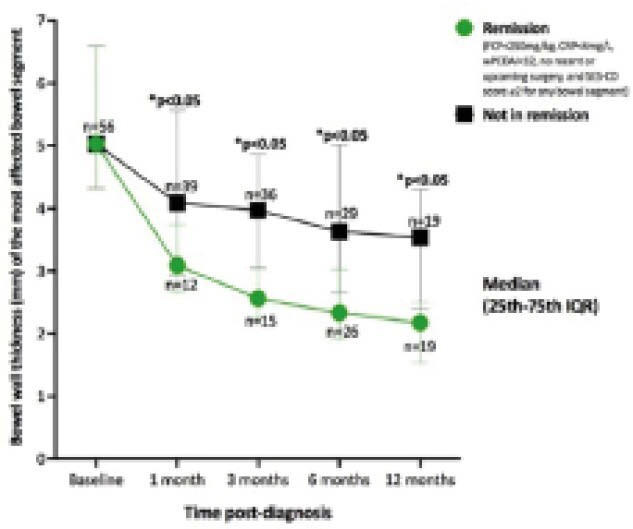

**Conclusion(s):**

TABUS had significant correlations with clinical, biochemical, and endoscopic markers of Crohn’s disease activity in pediatric patients over one year. Bowel wall thickness of the most affected bowel segment is a helpful measurement, becoming significantly thinner as soon as 1-month post-diagnosis in those who obtain remission. Baseline findings of bowel narrowing and upstream dilation increase the odds and relative risk of needing surgery in the first year.

**Please acknowledge all funding agencies by checking the applicable boxes below:**

None

**Disclosure of Interest:**

None Declared

